# Biodegradable Chitosan Films Incorporated with β-Cyclodextrin Microcapsules Loaded Clove with Essential Oil for Table Grape Preservation

**DOI:** 10.3390/foods15172957

**Published:** 2026-08-22

**Authors:** Cuixia Yang, Penghui Wei, Zhaotong Duan, Tinghui Duan, Mina Nan, Huali Xue, Yang Bi, Yan Yin

**Affiliations:** 1 College of Science, Gansu Agricultural University, Lanzhou 730070, China; 18419087402@163.com (C.Y.); 15101938641@163.com (P.W.); 19996073552@163.com (Z.D.); 19119399242@163.com (T.D.); nanmn@gsau.edu.cn (M.N.); 2 College of Food Science and Engineering, Gansu Agricultural University, Lanzhou 730070, China; biyang@gsau.edu.cn; 3 Agricultural Science and Technology Research Extension Center of Lanzhou, Lanzhou 730000, China; 17361646668@163.com

**Keywords:** chitosan, β-cyclodextrin, clove essential oil, microcapsules, grape preservation

## Abstract

Postharvest spoilage of fresh fruits demands efficient bio-based packaging films. Here, chitosan/gelatin (CG) films were incorporated with β-cyclodextrin-encapsulated clove essential oil microcapsules (β-CD@CEO MCs) at varying loadings. The results suggested that CEO encapsulation occurred within β-CD cavities and hydrogen-bond binding of MCs to the CG matrix. At 0.4% MCs, the composite film showed 60.11% higher tensile strength, excellent UV shielding, lower water vapor transmission rate, and strong antioxidant activity (DPPH 89.6%, ABTS 97.1), along with 58.21% biodegradation after 16 days of soil burial. In vitro release studies revealed a pH-responsive sustained release profile of CEO from the composite films, with faster release under acidic conditions (98.5% at pH 3.5 after 72 h) compared to neutral conditions (87.2% at pH 7.0), indicating the potential for targeted release on the weakly acidic grape surface. The film also exhibited significant antimicrobial effects against *Botrytis cinerea*, *Penicillium gladioli*, *Staphylococcus aureus*, and *Escherichia coli*. In table grape preservation, CG/MCs-0.4 film effectively delayed decay, reduced weight loss by 38.79%, maintained firmness and color, and preserved higher levels of soluble solids, titratable acidity, reducing sugars, and vitamin C compared to polyethylene packaging and untreated controls.

## 1. Introduction

Food packaging is a critical component in ensuring the safety of the food supply chain, extending shelf life, and minimizing economic losses. By preventing or blocking direct contact between food and its surroundings, it effectively slows down spoilage and deterioration [1]. Conventional petroleum-based plastic packaging presents serious challenges, including severe plastic pollution (it is referred as “white pollution”) and resource depletion, owing to its non-biodegradable nature and reliance on fossil resources. In addition, the degradation of these materials into small particles enables them to enter the human food chain, which in turn may exert detrimental effects on human health [2]. Therefore, the development of renewable, biodegradable, and functionally active green packaging materials has become a key research priority at the interface of food science and materials science. Among these, the edible or biodegradable films based on natural polymers manifest great application promise due to their environmental friendliness, biocompatibility, and potential as carriers for active compounds [3].

Among natural polymers, chitosan (CS), as a cationic polysaccharide, is derived from chitin deacetylation, which exhibits desirable properties, including excellent film-forming ability, biodegradability, biocompatibility, and broad-spectrum antimicrobial activity [4]. Gelatin (GL), derived from partial hydrolysis of collagen, exhibits excellent film-forming ability, transparency, gas barrier properties, and biodegradability [5]. However, Chitosan-based films are brittle, lack adequate flexibility, and exhibit poor solubility under neutral or alkaline conditions. Gelatin-based films have relatively low mechanical strength, insufficient thermal stability, and a marked tendency to absorb water and swell. Therefore, blending chitosan with gelatin forms a composite film matrix with a denser structure and superior overall performance, driven by electrostatic interactions between amino and carboxyl groups and synergistic hydrogen bonding. This matrix provides an ideal platform for subsequent functional modification [6]. Nevertheless, compared with dedicated antimicrobial agents, CS/GL-based films exhibit relatively modest antimicrobial activity. Additionally, they manifest certain shortcomings in thermal stability and mechanical robustness relative to conventional petroleum-based plastics [7]. Thus, incorporating natural active compounds with antimicrobial and antioxidant properties is an effective approach to enhance the performance of these active films [8]. These natural active compounds are incorporated into bio-based films, where they are released in a controlled manner to continuously interact with the food or its surrounding environment, thereby blocking external interference and ensuring food quality and safety [9,10].

Clove essential oil (CEO), as its major active component of eugenol (>80% of the oil), not only exhibits strong inhibitory effects against a broad spectrum of foodborne pathogenic and spoilage microorganisms by suppressing the secretion of extracellular polysaccharides and proteins [11] but also possesses excellent antioxidant capacity, scavenging free radicals and delaying lipid and pigment oxidation [12]. Therefore, CEO exhibits both antimicrobial and antioxidant activities, holding considerable promise for postharvest preservation of fruits and vegetables [13]. However, as a typical plant-derived essential oil, CEO exhibits high volatility, poor thermal stability, and low water solubility. Moreover, its rapid release kinetics prevent long-term preservation. Even when directly incorporated into polymer films, essential oils face challenges such as high volatility, uneven distribution within the matrix, and susceptibility to light and heat, all of which significantly compromise the efficacy of bio-functional films [14]. Thus, achieving stable incorporation, homogeneous distribution, and controlled release of essential oils in polymer film matrices represents a critical challenge for the fabrication of efficient active packaging films.

Microencapsulation technology is an effective strategy to overcome the aforementioned challenges. By encapsulating a core material (such as essential oils) within a wall material to form microcapsules, this approach not only protects the core from environmental factors, reduces its volatility, and masks intense odors, but also enables sustained release [15]. Among available wall materials, β-cyclodextrin (β-CD) has a unique structure with a hydrophobic inner cavity and a hydrophilic outer surface. This architecture enables β-CD to form microcapsules with various hydrophobic molecules (such as essential oil components) through host–guest interactions, thereby improving the water solubility, stability, and bioavailability of the encapsulated compounds [16,17]. Research has shown that encapsulating essential oils in β-cyclodextrin (β-CD) to form microcapsules not only enhances oil stability and reduces volatilization, but also facilitates their homogeneous dispersion into hydrophilic polymer matrices. This approach improves the microstructure and physical properties of the resulting composite films and enables sustained release profiles that can be diffusion-controlled or environmentally responsive [18]. In particular, β-CD encapsulation can enable controlled and sustained release of essential oil components, which is critical for maintaining long-term antimicrobial and antioxidant activity during food storage. Although β-CD encapsulation of essential oils in chitosan/gelatin films has been reported [14,17], the pH-responsive release behavior of such systems, particularly with quantitative kinetic modeling and its correlation with fruit preservation performance, has not been systematically investigated. This sustained-release function is especially vital for highly perishable berry fruits exemplified by table grapes.

The table grape, as a typical berry fruit, has few epidermal cell layers, a thin cuticle and wax layer, and abundant natural surface openings. Combined with vigorous respiration, severe water loss, and susceptibility to pathogen infection during postharvest storage, these factors collectively make grape fruits highly prone to decay and spoilage [19]. Consequently, developing preservation technologies that effectively suppress postharvest microbial decay, reduce oxidative damage, and minimize water loss in berry fruits is of particular significance [20]. By combining the physical barrier properties of a chitosan/gelatin composite film with the antimicrobial and antioxidant functionalities of β-CD@CEO microcapsules, a novel active packaging film can be engineered. This strategy offers great promise as an effective and environmentally friendly approach for postharvest grape preservation. However, the specific effects of β-CD@CEO microcapsule loading on the overall performance of such composite films remain unclear.

To address this gap, we systematically investigated the pH-responsive release behavior and integrated functional performance of chitosan/gelatin composite films incorporated with β-CD@CEO microcapsules at varying loadings for table grape preservation. Most previous studies have focused on either the formulation of chitosan/gelatin films or the incorporation of essential oils in free form, without addressing the controlled release functionality offered by β-CD encapsulation. Moreover, the effect of microcapsule loading concentration on the combined mechanical, barrier, antimicrobial, antioxidant, and preservation properties has not been systematically compared. Therefore, we prepared CG composite films with varying β-CD@CEO MC loadings (0.1%, 0.2%, and 0.4%) and comprehensively evaluated their structure, physicochemical properties, antimicrobial activity, antioxidant capacity, CEO release behavior, and preservation performance on table grapes. Specifically, this work provides (i) a detailed investigation of the release mechanism and pH-responsive behavior of CEO from β-CD microcapsules within a chitosan/gelatin film matrix, (ii) a comparative evaluation of the effects of microcapsule loading on integrated functional performance, and (iii) direct validation of the preservation efficacy on table grapes over 15 days of storage.

## 2. Materials and Methods

### 2.1. Materials

Chitosan (degree of deacetylation ≥95%, viscosity 100–200 mPa·s) was purchased from Shanghai Aladdin Biochemical Technology Co., Ltd. (Shanghai, China). Gelatin (analytical grade) was obtained from Laiyang Shuangshuang Chemical Co., Ltd. (Laiyang, China). Clove essential oil (CEO, biological reagent grade, 85% purity) was purchased from Shanghai Yuanye Bio-Technology Co., Ltd. (Shanghai, China). Glycerol (analytical grade), anhydrous ethanol (analytical grade), and glacial acetic acid (analytical grade) were purchased from Tianjin Fuyu Fine Chemical Co., Ltd. (Tianjin, China) and Chengdu Kelon Chemical Co., Ltd. (Chengdu, China), respectively. β-Cyclodextrin (β-CD, purity >97%) was obtained from Solarbio. Kyoho grapes were purchased from a local market (Taohai Market, Anning District, Lanzhou City) and selected for uniformity in size, shape, and ripeness, with no signs of pests, diseases, or mechanical damage.

### 2.2. Methods

#### 2.2.1. Preparation of β-CD@CEO Microcapsules (β-CD@CEO MCs)

β-CD@CEO MCs were prepared using a method modified from that described by Jiang et al. [21]. First, 6.0 g of β-CD was dissolved in 30 mL of distilled water with stirring to form a supersaturated solution. Then, 1 g of CEO was dissolved in 60% ethanol at a volume ratio of 1:7 (CEO: ethanol).

The supersaturated β-CD solution was ultrasonicated at 45 °C for 30 min (65 W of ultrasonic power). During ultrasonication, the CEO–ethanol solution was slowly added dropwise to the β-CD solution to form a microencapsulated emulsion. After incubation in a 45 °C water bath for 30 min, the mixture was cooled to room temperature and then refrigerated at 4 °C for 24 h. The resulting suspension was vacuum-filtered, and the precipitate was collected. The precipitate was washed successively with distilled water and anhydrous ethanol to remove CEO adsorbed on the β-CD surface, and then dried in an oven at 45 °C to obtain the β-CD@CEO MC product.

#### 2.2.2. Preparation of Chitosan/Gelatin/β-CD@CEO MC (CG/β-CD@CEO MCs) Composite Films

Composite films were prepared using a method modified from that reported by Yin et al. [22]. First, chitosan (1.0% *w*/*v*) was dissolved in glacial acetic acid (1% *v*/*v*) under magnetic stirring at 45 °C for 1 h to obtain the chitosan matrix. Glycerol (30% *w*/*w* based on chitosan dry weight) was added as a plasticizer, and the mixture was stirred thoroughly. Then, gelatin (1.0% *w*/*v*) was dissolved in distilled water under magnetic stirring at 45 °C for 1 h. Glycerol (30% *w*/*w* based on gelatin dry weight) was added to the gelatin matrix and stirred thoroughly to ensure complete dispersion. The two matrix solutions were then mixed at a 1:1 ratio to obtain the chitosan/gelatin composite film-forming solution.

β-CD@CEO MCs were added as a dry powder to the composite film solution at concentrations of 0.1%, 0.2%, and 0.4% (*w*/*v*) without the use of any organic solvent. The mixture was magnetically stirred at 45 °C for 45 min, then ultrasonically defoamed. Subsequently, 25 mL of the composite solution was poured onto a plastic Petri dish (90 mm × 90 mm) and allowed to dry naturally in a clean environment at room temperature (25 °C) for approximately 48 h. The Petri dishes were covered with perforated lids during drying to prevent dust and other particle contamination while allowing proper air circulation. The dried films were removed from the dishes and designated as CG/MCs-0.1, CG/MCs-0.2, and CG/MCs-0.4, respectively. They were then equilibrated for 2 days in a chamber (Model HWS-250, Shanghai Jinghong Experimental Equipment Co., Ltd., China) that maintained a temperature of 25 °C and 50% relative humidity (RH) before use in subsequent experiments. The CK group (CG film without MCs) was prepared using the identical solvent system (1% acetic acid and water) and drying procedure.

### 2.3. Characterization of β-CD@CEO MCs and Composite Films

The characterization methods for β-CD@CEO MCs and the CG/β-CD@CEO composite films (FTIR, XRD, TGA, and SEM) are described in detail in the Supplementary Materials (Text S1).

#### Determination of Drug Loading (DL) and Encapsulation Efficiency (EE)

The drug loading and encapsulation efficiency of β-CD@CEO microcapsules were determined using UV-visible spectrophotometry, according to the method described by Huang et al. [23] with minor modifications. Briefly, 100 mg of dried microcapsules was accurately weighed and dispersed in 10 mL of anhydrous ethanol, followed by ultrasonication for 30 min. The mixture was then diluted to a final volume of 35 mL. After centrifugation at 4000 r/min for 10 min, the supernatant was collected and its absorbance was measured at 280 nm. The eugenol concentration in the extract was calculated using a standard curve (y = 0.0273x − 0.0069, R^2^ = 0.9998). DL and EE were calculated using the below formulas, and all results are expressed as eugenol equivalents. Since eugenol is the major active component of CEO (>85%) and served as the reference standard for quantification, the eugenol-equivalent loading provides a reliable measure of the active CEO content in the microcapsules.

(1) $$DL\left(\%\right)=\frac{C\times D\times V}{M_{sample}}\times 100\%$$

(2) $$EE\left(\%\right)=\frac{DL\times M_{total\;MCs}}{M_{input\;oil}}\times 100\%$$

where *C* is the diluted concentration, *D* is the dilution factor, *V* is the extraction volume, and *M*_sample_, *M*_total MCs_, and *M*_input oil_ are the masses of the sample, total microcapsules, and initial oil, respectively.

### 2.4. Properties of CG/β-CD@CEO Composite Films

#### 2.4.1. Thickness and Mechanical Properties

Film thickness was measured using a digital micrometer (accuracy: 0.001 mm; Shengtaixin Electronic Technology Co., Ltd., Huzhou, China). For each film sample, thickness was measured at five randomly selected locations, and the average value was used for calculation Tensile strength (TS) and elongation at break (EB) were determined with a universal testing machine (MTS Industrial Systems Co., Ltd., Shanghai, China). The films were cut into rectangular strips (15 mm × 60 mm) and tested at an initial grip separation of 40 mm and a crosshead speed of 10 mm/min. The following equations were used for calculations [24]:

(3) $$TS\left(MPa\right)=\frac{F}{L\times M}$$

(4) $$EB\left(\%\right)=\frac{L_{1}-L_{0}}{L_{0}}\times 100$$

where F (N) is the maximum force, L (mm) is the thickness, M (mm) is the width, L_1_ (mm) is the final length of the film at break, and L_0_ (mm) is the initial length of the film.

#### 2.4.2. Color and Opacity Properties

The L* (lightness), a* (redness), and b* (yellowness) values of the films were measured using a colorimeter (SC-10+, Shenzhen, China).

Film opacity was measured using a UV-Vis spectrophotometer (UV-1780, Shimadzu, Japan). The film was cut into 10 mm × 40 mm rectangles and attached to the inner wall of a quartz cuvette. Absorbance was measured at 600 nm, and opacity was calculated using Equation (5):

(5) $$Opacity=\frac{A_{600\;nm}}{L}$$

where A_600_ is the absorbance measured at 600 nm, and L is the film thickness (mm).

#### 2.4.3. Moisture Content (MC) and Water Solubility (WS)

The film was cut into 20 mm × 20 mm squares. The initial mass (m_1_) was recorded, and the mass (m_2_) was measured after drying at 105 °C for 24 h. The dried film was then immersed in 50 mL of water for 2 h. After blotting the surface moisture, the film was dried again, and the mass (m_3_) was measured. Moisture content and water solubility were calculated using Equations (6) and (7), respectively [25]:

(6) $$MC\left(\%\right)=\frac{m_{1}-m_{2}}{m_{1}}\times 100$$

(7) $$WS(\%)=\frac{m_{2}-m_{3}}{m_{2}}\times 100$$

#### 2.4.4. Water Vapor Permeability (WVP)

The water vapor permeability (WVP) of the composite film was measured using the W3/030 Water Vapor Permeability Tester (Jinan Languang Electromechanical Technology Co., Ltd., Jinan, China) at 38 °C and 90% relative humidity (RH).

#### 2.4.5. Light Transmittance Properties

Light transmittance properties of the films were evaluated by measuring transmittance in the 200–800 nm range using a UV-Vis spectrophotometer.

#### 2.4.6. Water Contact Angle (WCA)

Water contact angle (WCA) of the films was measured using a CA200 automatic optical contact angle meter (North Precision Instruments Co., Ltd., Dongguan, China). A 2 μL drop of distilled water was placed onto the film surface and immediately photographed. WCA was measured at three randomly selected points per sample.

### 2.5. Antioxidant Activity Assays

Radical scavenging activity of the composite films was evaluated using DPPH and ABTS assays. The specific procedure was based on the method described by Qin et al. [26], with slight modifications. The scavenging activities were calculated using Equations (8) and (9), respectively:

(8) $$DPPH\left(\%\right)=1-\frac{A_{s}-A_{b}}{A_{c}}\times 100$$

(9) $$ABTS(\%)=1-\frac{A_{s}-A_{b}}{A_{c}}\times 100$$

where A_s_ is the absorbance of the sample, A_b_ is the absorbance of the sample background, and A_c_ is the absorbance of the blank control.

### 2.6. Biodegradability Assessment

The biodegradability test was conducted by the indoor soil burial method described by Tai et al. [27]. The films were cut into 25 mm × 25 mm squares and dried to constant weight, and their initial mass was recorded. Each film sample was wrapped in a 500-mesh nylon net and buried in soil at a depth of 10 cm. The soil moisture was maintained at 75% using the gravimetric method: the container with soil was weighed daily, and deionized water was added as needed to maintain the initial weight, ensuring constant moisture throughout the experiment. The temperature was maintained at 25 ± 2 °C. At 0, 4, 8, 12, and 16 days, the samples were retrieved and weighed. The biodegradation rate was calculated using Equation (10):

(10) $$Biodegradation\left(\%\right)=\frac{W_{0}-W_{i}}{W_{0}}\times 100$$

where W_0_ is the initial mass (g), and Wi is the mass measured on days 0, 4, 8, 12, and 16.

### 2.7. Antimicrobial Properties

#### 2.7.1. Antifungal Properties

The antifungal activity of the composite films was evaluated using the poisoned agar method [28], with detailed procedures provided in the Supplementary Materials (Text S2).

#### 2.7.2. Antibacterial Properties

Antibacterial activity was evaluated using the disk diffusion method (Kirby–Bauer method), following the procedure described by Wang et al. [29], with detailed procedures provided in the Supplementary Materials (Text S2).

Both the poisoned agar and disk diffusion assays were conducted using the film-forming solutions (CG/MCs with varying MC loadings). The same solutions were applied as coatings on grape surfaces in the preservation experiments.

### 2.8. In Vitro Release Assay of Composite Films

The release experiment was performed according to the method of Adjali et al. [30] with slight modifications. The equilibrated CG/MCs-0.4 composite films were cut into small pieces of approximately 0.5 cm × 0.5 cm, and 200 mg of the film fragments were accurately weighed and placed into 250 mL conical flasks containing 100 mL of release medium (deionized water at pH 7.0 or acetate buffer at pH 3.5, which was selected to mimic the natural weakly acidic microenvironment of grape surfaces). Blank CG films (200 mg) without microcapsules were used as controls to subtract the background absorbance of the film matrix. Each group consisted of three parallel replicates. The flasks were incubated in a thermostatic shaker at 25 ± 1 °C with constant shaking at 180 r/min. At predetermined time intervals (0.5, 1, 2, 4, 6, 8, 12, 24, 48, and 72 h), 2 mL aliquots of the release medium were withdrawn and immediately replaced with an equal volume of fresh pre-warmed medium to maintain a constant total volume. The collected samples were filtered through a 0.45 μm syringe filter, and the absorbance was measured at 280 nm. The concentration of eugenol in the release medium was calculated using a eugenol standard curve (y = 0.0273x − 0.0069, R^2^ = 0.9998). The cumulative release amount (Q_t_, μg/mg) and cumulative release rate (R_t_, %) were calculated according to the following equations:

(11) $$Q_{t}(\mu g/mg)=\frac{V\cdot C_{t}+v\cdot \sum_{i=1}^{n-1}C_{i}}{M}$$

(12) $$R_{t}\left(\%\right)=\frac{Q_{t}}{Q_{max}}\times 100\%$$

where V is the total volume of the release medium (100 mL), v is the sampling volume (2 mL), C_t_ is the eugenol concentration at time t (μg/mL), M is the mass of the film sample (200 mg), and Q_max_ is the theoretical maximum cumulative release amount (3.83 μg/mg), calculated based on the measured drug loading of the composite film (0.383%, expressed as eugenol equivalents. as detailed in the Supplementary Materials Text S3 and Table S1).

### 2.9. Applications for Table Grape Preservation Experiments

#### Table Grape Fruit Treatment and Measurement of Fruit Quality Parameters

The preparation and treatment of table grapes are described in detail in the Supplementary Materials (Text S4). In brief, based on the comprehensive characterization results, the CG/MCs-0.4 formulation was selected for the grape preservation test based on a balanced consideration of its mechanical, barrier, antioxidant, and antimicrobial performance among the three MC loadings tested. Grapes were divided into three groups: control (CK), polyethylene (PE) packaging, and CG/MCs-0.4 coating. The coating thickness was estimated to be 27.9 ± 0.4 μm, as determined on glass slides using a digital micrometer. All samples were stored at 4 °C and 90% relative humidity for 15 days, and quality indicators were measured every 3 days.

The weight loss rate of the grapes was calculated using Equation (13):

(13) $$Weight\;loss\;rate\left(\%\right)=\frac{Original\;weight-The\;weight\;after\;storage}{Original}\times 100$$

Total soluble solids (TSSs) were measured with a sugar–acidity meter (WZ-103). Fruit hardness was determined using a GY-4 hardness tester. Color changes were recorded with a colorimeter (SC-10+).

The respiration rate was calculated according to the method of Grudzinska et al. [31] with minor modifications. The respiration rate was calculated using Equation (14):

(14) $$Respiration\;rate(mg{CO}_{2}\cdot {kg}^{-1}\cdot h^{-1})=\frac{V\times \left(C_{1}-C_{2}\right)\times 44}{m\times t\times 22.4}$$

where V is the total volume of the sealed container (L), C_1_ and C_2_ are the CO_2_ volume fractions (%) measured by the portable analyzer before and after time t, respectively, m is the mass of the grapes (kg), and t is the measurement duration (h).

Titratable acidity was determined using the method described by Lan et al. [32]. The vitamin C (VC) content was determined according to the method described by Liang et al. [33]. The reducing sugar content in the fruit was determined using the 3,5-dinitrosalicylic acid method, following the procedure described by Lam et al. [34].

### 2.10. Statistical Analysis

All experiments were performed with at least three biological replicates. Results are expressed as mean ± standard deviation (SD). Data were analyzed by one-way ANOVA using SPSS 27.0, and significant differences (*p* < 0.05) are indicated by different letters. Graphs were prepared using Origin 2022, and schematic diagrams were created with BioGDP.com.

## 3. Results and Discussion

### 3.1. Characterization of β-CD@CEO Microcapsules

**Figure 1:**
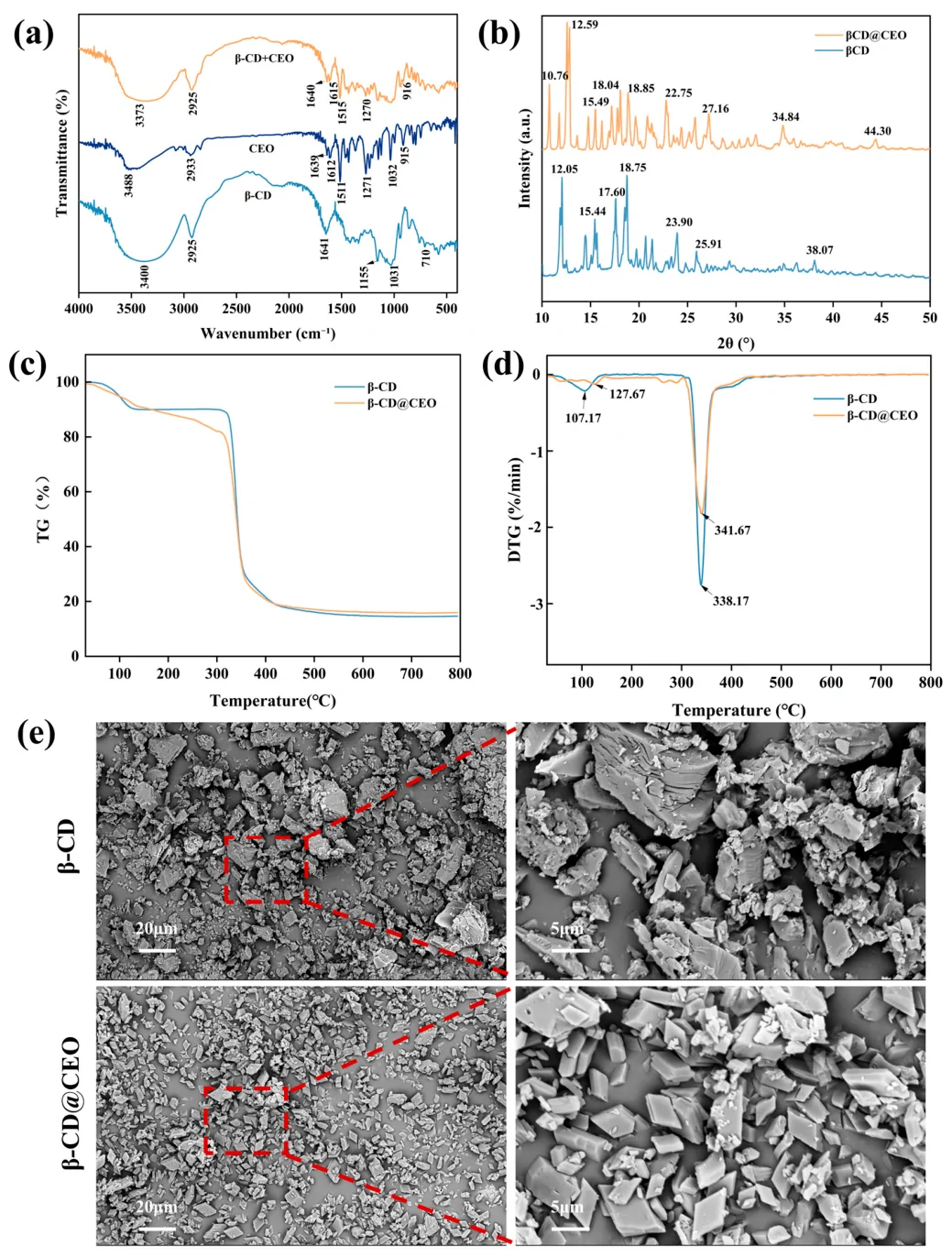
FTIR spectra (**a**), X-ray diffraction (XRD) patterns (**b**), thermogravimetric (TG) (**c**) and derivative thermogravimetric (DTG) (**d**) curves, and scanning electron microscopy (SEM) image (**e**) of β-CD, CEO, and β-CD@CEO MCs.

The FTIR spectrum of β-CD (Figure 1a) shows a broad, intense O–H stretching peak at 3400 cm^−1^, a C–H vibration peak at 2925 cm^−1^, an O–H bending peak of adsorbed water at 1641 cm^−1^, and glycosidic backbone vibration peaks at 1031 cm^−1^ and 710 cm^−1^ [35]. The CEO spectrum shows peaks at 3488 cm^−1^ (phenolic O–H stretching), 2933 cm^−1^ (C–H vibration), 1511 cm^−1^ (aromatic ring skeletal vibration), and 1271 cm^−1^ (C–O stretching of aryl-alkyl ether) [36]. In the infrared spectrum of the β-CD@CEO microcapsules, the characteristic peak of the CEO aromatic ring (1511 cm^−1^) remained visible, while some peaks shifted from 1611 cm^−1^ to 1615 cm^−1^ (the CEO peak). The peak shifts and the presence of CEO characteristic bands in the microcapsule spectrum are indicative of host–guest interactions between CEO and β-CD [17] consistent with the formation of an inclusion complex rather than simple physical adsorption, as also observed by Yahiya et al. [37].

The XRD pattern of β-CD (Figure 1b) shows sharp characteristic diffraction peaks at 2θ = 12.05°, 18.75°, and 23.90°, corresponding to the crystal structure formed by ordered stacking of β-CD molecules, consistent with Wang et al. [38]. In the XRD patterns of β-CD@CEO MCs, new peaks appeared at 2θ = 10.76°, 22.75°, 27.16°, and 34.84°, while the intensities of the original peaks shifted significantly, suggesting structural modifications of the β-CD lattice upon CEO incorporation [39]. The microcapsules retained crystalline diffraction peaks rather than exhibiting complete amorphization. This can be attributed to two factors. First, the good size match between eugenol and the β-CD cavity did not severely disrupt the overall crystal lattice. Second, the mild saturated solution method preserved partial crystalline order. These observations are consistent with similar β-CD-essential oil systems prepared by coprecipitation [21]. Inclusion complexes of essential oils with cyclodextrins are known to adopt a channel-type crystal packing mode with high crystalline order in the solid state [14]. The observed new diffraction peaks, together with the intensity changes, are consistent with the formation of the inclusion complex.

The TGA/DTG results (Figure 1c,d) show that the decomposition process consists of two stages: low-temperature water removal and high-temperature framework decomposition. During the dehydration stage (30–120 °C), β-CD exhibited significant mass loss (10–12%), with a small DTG peak at 107.17 °C corresponding to removal of surface-adsorbed water. In contrast, β-CD@CEO showed more gradual mass loss, and its DTG dehydration peak shifted to 127.67 °C, attributed to hydrophobic clove essential oil replacing water molecules inside the β-CD cavities. During the backbone decomposition stage (300–400 °C), the main decomposition peak of β-CD appeared at 338.17 °C, with high DTG intensity and rapid weight loss. For β-CD@CEO, the DTG peak intensity decreased because CEO molecules were filled the β-CD cavities, providing structural support to the backbone and delaying thermal decomposition, thus suggesting enhanced thermal stability of the microcapsules [17]. The significant differences in thermal decomposition in the two stages described above suggest that the association of CEO with the β-CD cavity substantially altered the thermal decomposition behavior of β-CD.

The SEM image (Figure 1e) revealed that β-CD particles were irregularly shaped with rough, uneven surfaces, a morphology typical of mechanically dispersed crystals after aggregation and likely associated with the oven-drying process, which promotes intermolecular hydrogen bonding and dense particle packing [40]. The β-CD@CEO microcapsules exhibited a regular rhombohedral shape with a smooth, dense surface and reduced fracturing surfaces, which can be attributed to the incorporation of CEO into the β-CD matrix and the filling of crystal pore defects. Regarding size distribution, the β-CD@CEO particles were finer, which is consistent with the general trend that particles become finer and more dispersed after β-CD encapsulates hydrophobic guests, and in agreement with the results reported by Stancu et al. [41].

Collectively, the FTIR, XRD, TGA, and SEM analyses provide multiple lines of evidence supporting the formation of β-CD@CEO inclusion complexes. While these techniques are informative for assessing bulk structural and thermal properties, direct elucidation of the host–guest inclusion structure at the molecular level would benefit from additional techniques such as DSC or NMR in future studies.

### 3.2. DL and EE of β-CD@CEO Microcapsules

The drug loading and encapsulation efficiency of β-CD@CEO microcapsules are summarized in Table 1. The average drug loading was 5.96 ± 0.26%, and the average encapsulation efficiency was 38.67 ± 1.68% (both expressed as eugenol equivalents). The encapsulation efficiency is influenced by process parameters such as wall-to-core ratio, temperature, and ultrasonication time, and falls within the typical range of 21–98% for β-CD-encapsulated essential oils [23,41]. These results indicate that the ultrasonic-assisted coprecipitation method effectively encapsulated clove essential oil in β-CD, and the obtained microcapsules exhibited satisfactory drug loading.

### 3.3. Characterization of CG/β-CD@CEO Composite Films

**Figure 2:**
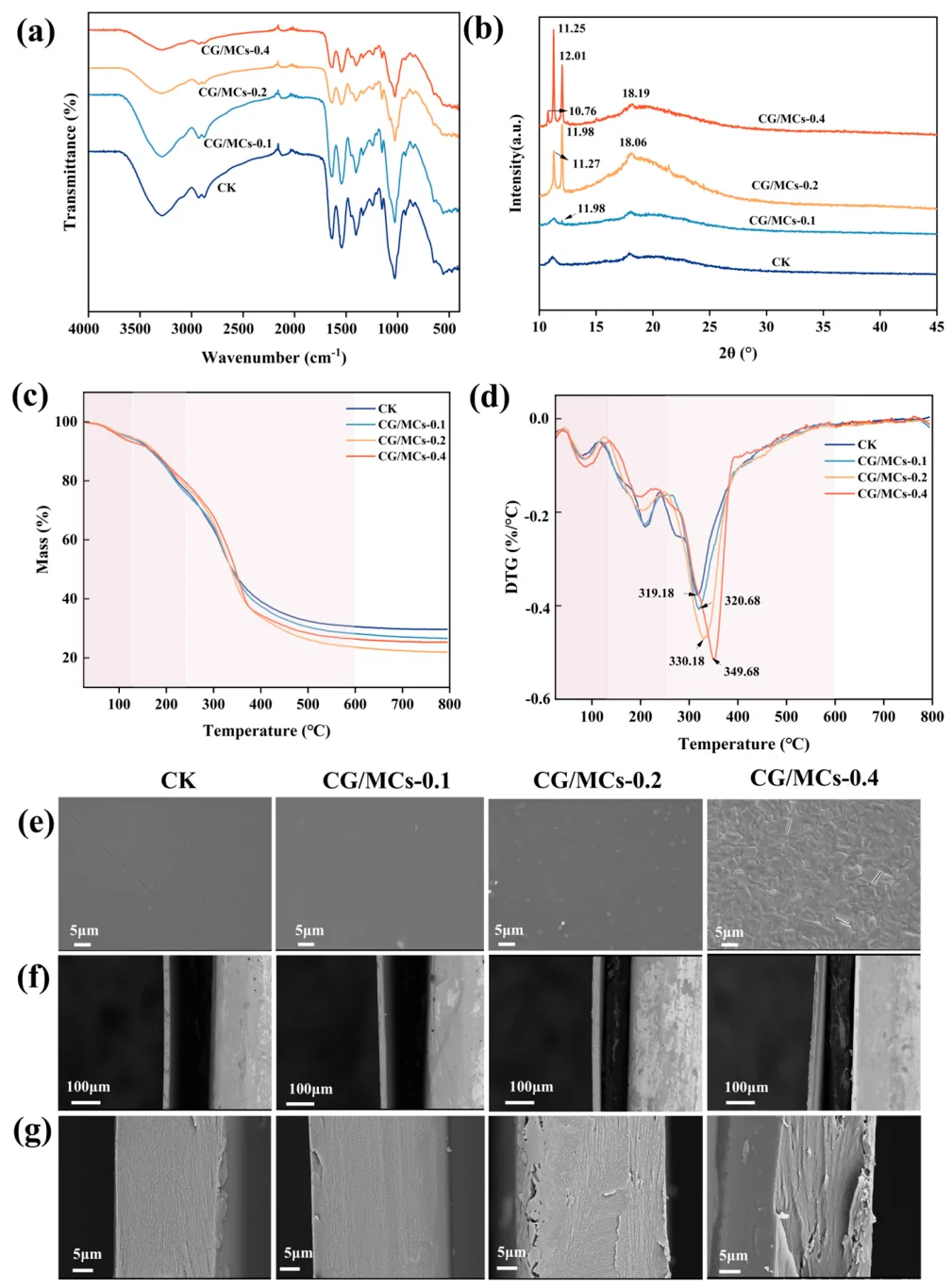
FTIR spectra (**a**), XRD patterns (**b**), TG and DTG curves (**c**,**d**), and SEM images of the surface (**e**) and cross-sections (**f**,**g**) of composite films containing different concentrations of β-CD@CEO MCs. The different shaded colors in (**c**,**d**) represent different thermal decomposition stages.

The FTIR spectra of the composite films containing different concentrations of β-CD@CEO MCs were generally consistent with those of the CG film (Figure 2a), suggesting that no significant chemical reaction occurred during film formation. In the hydroxyl and amino stretching region (3000–3500 cm^−1^), the absorption peak of the composite films shifted from 3295 cm^−1^ to 3284 cm^−1^ (red shift) and became notably broader. This change is consistent with the formation of strong intermolecular hydrogen bonds between the β-CD@CEO MCs and the CG matrix, which reduced the vibration energy of the O–H/N–H bonds [42]. Furthermore, the characteristic absorption regions such as the amide I (~1650 cm^−1^) and amide II (~1550 cm^−1^) bands, the peak shapes of the composite film broadened and their intensities weakened, further supporting hydrogen bonding interactions. These results suggest that the incorporation of β-CD@CEO MCs is associated with the CG film matrix via hydrogen bonding, forming a more compact molecular network.

Figure 2b shows the XRD patterns of composite films containing different concentrations of β-CD@CEO MCs and the CG film. The CK group exhibited characteristic diffraction peaks of chitosan/gelatin at 2θ = 11.98°, corresponding to its amorphous-to-crystalline phase transition. In contrast, all composite films containing β-CD@CEO MCs showed characteristic peaks of β-CD (e.g., 2θ = 10.76°, 11.27°, and 12.01°), and the peak intensity increased with increasing microcapsule concentration, indicating that β-CD@CEO MCs were stably dispersed in the composite film matrix, with the loading amount increasing proportionally to the added concentration. Concurrently, the characteristic peak of the CK film at 11.98° was masked by the β-CD peak at 12.01°. This is attributed to hydrogen bonding between the hydroxyl groups of β-CD and the amino and hydroxyl groups of the CG film. These interfacial interactions not only preserve the crystalline stability of the β-CD@CEO MCs, but also weaken the self-crystallization ability of the matrix molecules, thereby potentially enhancing the interfacial bonding strength between the microcapsules and the matrix [43].

Thermal stability is a key indicator of film performance under different temperature conditions. The TGA/DTG results (Figure 2c,d) suggest that all films exhibited similar three-stage thermal decomposition curves. Films containing MCs demonstrated better thermal stability. The most significant decomposition stage occurred between 250 and 400 °C. The composite films containing 0.1%, 0.2%, and 0.4% MCs exhibited maximum thermal decomposition temperatures of 320.68, 330.18, and 349.68 °C, respectively, all were higher than that of the control group (319.18 °C). This improvement is attributed to CEO molecules filling the β-CD cavities and forming a skeletal support, which delays thermal decomposition and thus enhances the overall thermal stability of the composite film matrix [44].

SEM observation (Figure 2e–g) suggested that the CK group had a flat, dense surface and a continuous, pore-free cross-section, representing a typical uniform structure of polysaccharide-based films. Upon addition of 0.1% microcapsules, the film surface remained relatively smooth, with only slight protrusions observed in the cross-section. When the microcapsule concentration increased to 0.2%, more surface particles was observed, the cross-section became rougher, and localized protrusions emerged. At 0.4% microcapsules, rhombic MC crystals were uniformly distributed within the film, and the cross-section displayed micropores, rough edges, and a complex porous structure [45]. These observations are consistent with XRD findings and suggest that the addition of β-CD@CEO MCs may modulate the structure and properties of the composite films.

### 3.4. Physical and Chemical Properties of CG/β-CD@CEO Composite Films

#### 3.4.1. Color, Thickness, Opacity, UV Barrier Properties, and Mechanical Properties

Excellent optical properties of packaging materials can effectively protect food from UV-induced photooxidation [46]. The effects of adding different concentrations of β-CD@CEO MCs on the physicochemical properties of the composite films were investigated. The incorporation of β-CD@CEO MCs systematically altered the optical characteristics and physical structure of the films (Table 2). With increasing MC concentration, the lightness L* value decreased significantly, while the b* value increased markedly, resulting in a greater color difference. These optical changes were mainly attributed to the physical effect of MC addition, which disrupted the structural homogeneity of the film matrix and enhanced light scattering. Film thickness also showed a slight but significant linear increase with increasing MC, confirming its role as a solid filler [47].

**Figure 3:**
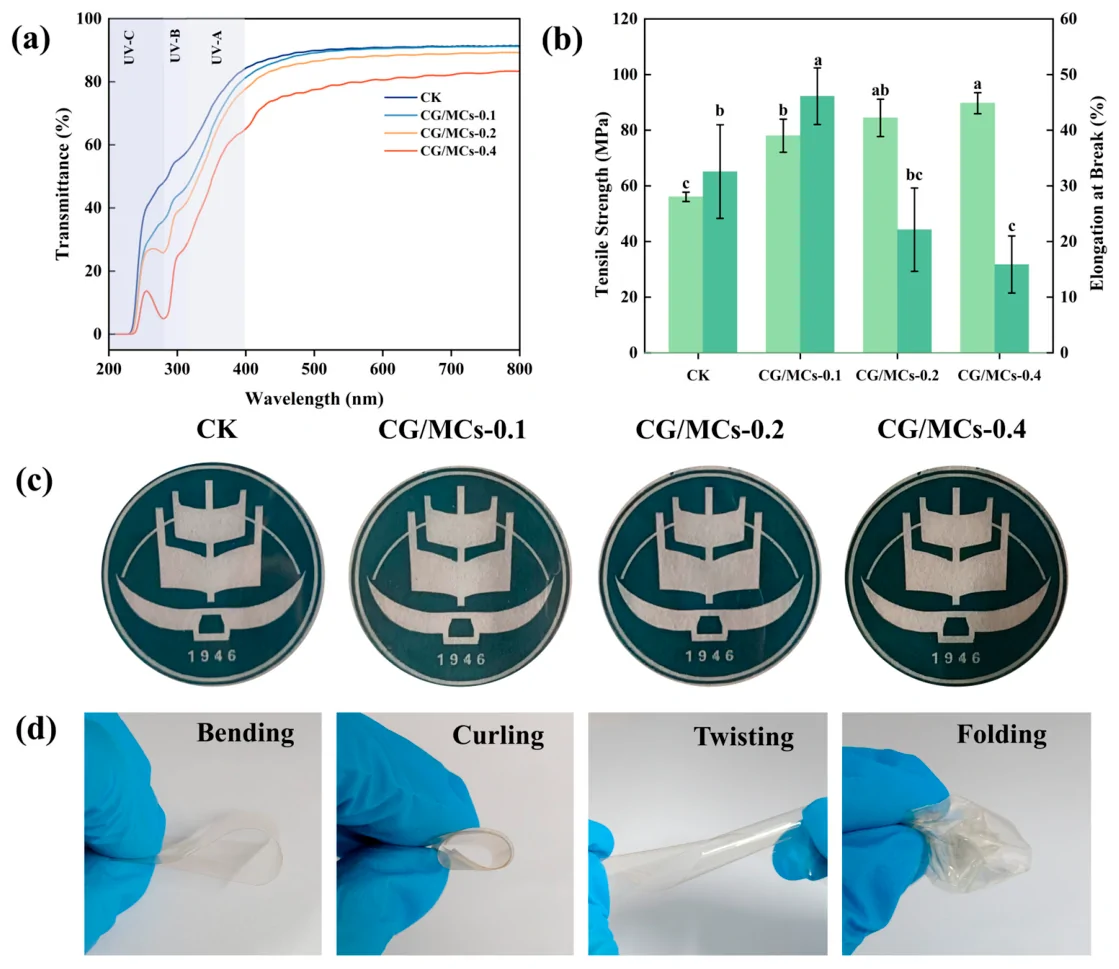
Light transmittance properties (**a**), tensile strength (TS) and elongation at break (EB) (**b**), color change (**c**), and bending, curling, twisting, and folding of the CG/MCs-0.4 film (**d**) of CG/MC composite films. Different letters indicate significant differences among groups (*p* < 0.05).

UV-visible transmission spectra were measured in the 200–800 nm range (Figure 3a). Different concentrations of MCs significantly modulated the barrier properties of the composite films. The CG film exhibited a transmittance of 30–70% in the UV region (200–400 nm), indicating its limited inherent UV-blocking capability. After MC addition, UV transmittance decreased significantly and further decreased with increasing MC concentration. Notably, when the MC was 0.4%, the T280 value of the composite film was only 4.93%, compared to 48.12% for the CG film. This indicates that the addition of MCs provides excellent UV-blocking performance at 280 nm. At least two mechanisms underlie this effect: selective absorption of low-wavelength UV radiation by phenolic compounds in clove essential oil [48] and light scattering by β-CD. Previous studies have also reported that films containing phenolic compounds exhibit lower UV-visible transmittance [46,49,50]. The results showed that the MC-incorporated composite films exhibited excellent UV-blocking performance.

Superior mechanical properties improve the durability and stress resistance of food packaging films during processing, transport, storage, and distribution [51]. As shown in Figure 3b, tensile strength (TS) exhibited a significant and sustained upward trend from the CK group to the CG/MCs-0.4 group, increasing from 56.03 MPa to 89.71 MPa (a 60.11% increase). This may be attributed to the formation of a dense intermolecular hydrogen-bond network between the numerous primary and secondary hydroxyl groups on the outer surface of β-CD molecules and the amino and hydroxyl groups of chitosan. This network effectively transfers stress and restricts polymer chain slippage, thereby increasing TS. FTIR results also confirmed this point. Yin et al. [22] suggested that the incorporation of cinnamaldehyde-tannic acid-zinc acetate nanoparticles into CS films improved the TS of the composite films EB increased at a low MC concentration (0.1%) and then decreased, as similar concentration-dependent mechanical behavior has been observed in other essential oil-incorporated chitosan/gelatin composite films [52]. In the CK group, EB was 32.57%; when 0.4% microcapsules were added, EB decreased to 15.17%, which was because at low concentrations, the hydrophobic components of the microcapsules exerted a plasticizing effect by shielding intermolecular hydrogen bonds, while at high concentrations, they acted as rigid fillers that restricted polymer segment movement and disrupted the biopolymer network structure [53]. SEM images also confirmed this point. Figure 3d showed that the CG/MCs-0.4 film could be randomly bent, curled, twisted, and folded, demonstrating its flexibility in practical applications.

#### 3.4.2. Moisture Content, Water Solubility, Water Vapor Permeability, Contact Angle

**Figure 4:**
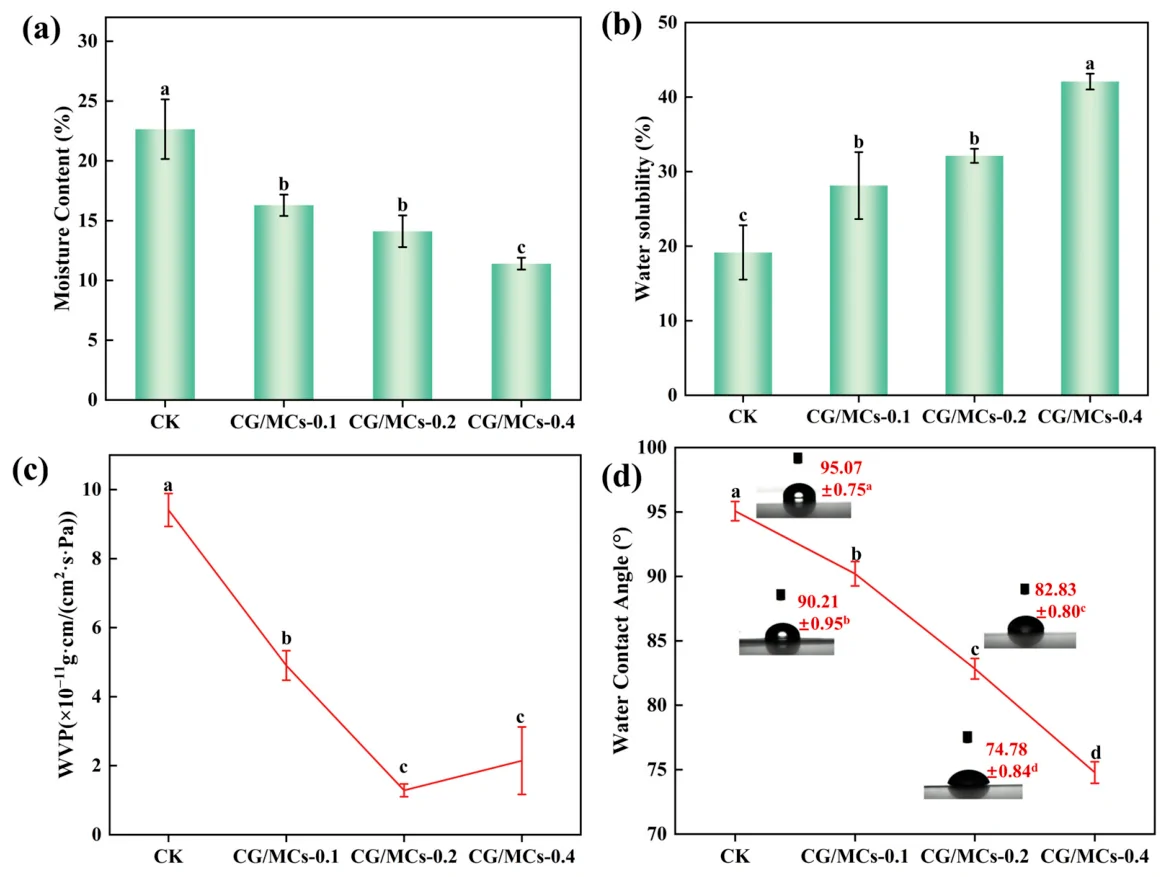
Moisture content (**a**), water solubility (**b**), water vapor permeability (**c**), and water contact angle (**d**) of CG/β-CD@CEO composite films. Different letters indicate significant differences among treatment groups (*p* < 0.05).

Figure 4 shows the moisture characteristics of films with and without MCs. As shown in Figure 4a, the CG film exhibited a higher moisture content (22.6%), which was attributed to the presence of numerous hydrophilic groups on chitosan molecules. The addition of MCs significantly reduced the water content of the films. For composite films containing 0.1%, 0.2%, and 0.4% MCs, the water content decreased to 16.3%, 14.1%, and 11.4%, respectively. This was because MCs filled some of the pores in the composite film, thereby reducing the ability of water molecules to occupy them [54].

For food packaging applications, films should exhibit good water resistance capacity. Figure 4b shows the water solubility of the composite films. The CG film exhibited the lowest water solubility (18.3%), while the addition of MCs increased its water solubility. This is attributed to the hydrophilicity of β-CD in MCs, which arises from its abundant hydroxyl groups and facilitates film mass loss upon immersion in water [13]. In addition, the incorporation of MCs may disrupt the hydrogen-bonding network between chitosan and gelatin, creating more free volume and allowing greater water uptake [39]. Therefore, incorporating MCs into the CG composite film does not prevent its partial dissolution upon contact with water.

WVP is one of the most important properties of a film, which influences moisture exchange between food and the environment. Composite films containing MCs exhibited lower WVP values. When the MC concentration increased from 0.1% to 0.2%, the WVP of the composite film decreased from 4.90 × 10^−11^ to 1.29 × 10^−11^ g·cm/(cm^2^·s·Pa) (Figure 4c). This reduction was mainly attributed to the tortuosity effect: the dispersed MCs acted as impermeable obstacles within the polymer matrix, forcing water vapor molecules to travel along longer and more convoluted diffusion pathways [55]. Furthermore, the presence of MCs in the film matrix restricted the mobility of polymer chains [39]. It is worth noting that, when the MC concentration was increased to 0.4%, the film WVP increased by 2.14 × 10^−11^ g·cm/(cm^2^·s·Pa). However, this value was still lower than that of the control (CK) film (9.41 × 10^−11^ g·cm/(cm^2^·s·Pa)). This effect was attributed to higher concentrations of MCs disrupting the uniform structure of the film matrix surface, making it easier for water molecules to pass through the film, which was consistent with the SEM results.

As shown in Figure 4d, the water contact angle decreased from 95.07° (CK) to 74.78° (CG/MCs-0.4) with increasing microcapsule concentration, indicating enhanced hydrophilicity of the film surface. This was because β-CD molecules are rich in hydroxyl groups, which increase the density of hydrophilic sites when exposed on the film surface. Simultaneously, the hydrophobic components of clove essential oil are encapsulated within the hydrophobic cavities of β-CD and cannot be directly exposed on the film surface, resulting in surface properties dominated by hydrophilic sites [56]; in addition, the introduction of MCs increased the roughness of the film surface, further reducing the contact angle.

The exposure of hydroxyl groups of β-CD on the film surface, together with increased surface roughness, reduced the water contact angle and enhanced surface wettability. Meanwhile, the dispersed MCs effectively decreased WVP and moisture content by increasing the tortuosity of diffusion pathways for water molecules and filling the free volume within the matrix. Since the tortuosity effect and pore-filling effect predominantly govern the improvement in bulk barrier properties, their contribution outweighs the influence of increased surface hydrophilicity; thus, the two effects do not constitute a contradiction. Nevertheless, the water resistance and solubility of the film still require further improvement, and future research should optimize the balance between MC loading and the overall hydrophobicity and structural stability of the film.

### 3.5. Antioxidant Activity

**Figure 5:**
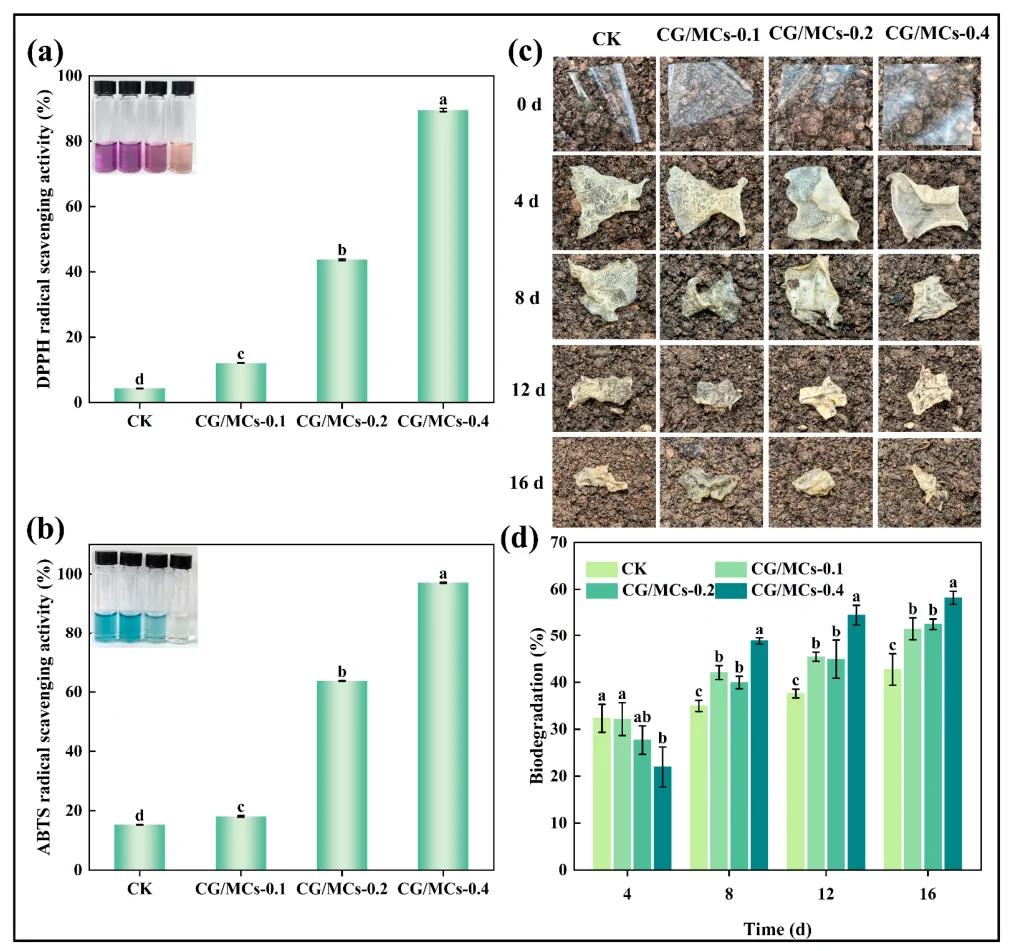
DPPH (**a**) and ABTS (**b**) radical scavenging activities of films in each group; visual changes (**c**) and biodegradation percentage (**d**) over 16 days under simulated soil burial conditions. Different letters indicate significant differences among groups (*p* < 0.05).

Oxidation reactions negatively affect the nutritional value and quality of food. Therefore, antioxidant capacity is a fundamental property of bioactive packaging materials. The radical scavenging activity of CG composite films containing different concentrations of β-CD@CEO MCs is shown in Figure 5. For DPPH radical scavenging (Figure 5a), the CG film showed only 4.3% activity. When the MC concentration was increased to 0.4%, the scavenging rate of CG/MCs-0.4 achieved 89.6%, which was 20.8 times higher than that of the CK group. For ABTS radical scavenging (Figure 5b), the CG film showed only 15.3% activity. As the MC concentration increased from 0.1% to 0.4%, the scavenging rate significantly improved, reaching 97.1% for CG/MCs-0.4, which was 6.3 times higher than that of the CK group. This effect is likely attributed to the inclusion complexation of β-CD stabilizing phenolic antioxidants (such as eugenol) in CEO, thereby enhancing the film’s ability to quench both hydrophilic and lipophilic radicals. This is consistent with the findings reported by Shen et al. [57].

### 3.6. Biodegradability Test

The biodegradation of the composite films was evaluated over 16 days under simulated soil burial conditions. All films showed progressive degradation, but the extent varied significantly (Figure 5c). By day 4, the CK group exhibited relatively rapid initial degradation, with a rate of 32.32% (Figure 5d). In contrast, the MC-containing films degraded more slowly initially; for instance, the CG/MCs-0.4 film had a degradation rate of only 21.94%. This difference may be explained by two complementary mechanisms: the dense microstructure of MC-containing films may have physically impeded water penetration and microbial invasion [43], and the burst release of CEO from β-CD microcapsules in the initial hours, as observed in our in vitro release experiment, likely delivered a relatively high concentration of antimicrobial compounds to the film surface and surrounding soil, temporarily suppressing the metabolic activity of soil microorganisms [58]. However, as the degradation period extended, the degradation rate of the CG/MCs-0.4 group increased sharply to 58.21% by day 16, while the CK group reached only 42.72%. Several factors may have contributed to this accelerated degradation: the gradual depletion of CEO would have removed the inhibitory effect; the released β-CD may have enhanced film hydrophilicity, promoting water penetration and enzymatic hydrolysis [4,12]; and β-CD could have served as an additional carbon source for microorganisms. These factors may have created a positive feedback loop, wherein the film became more porous and microbial penetration deepened, further accelerating degradation [15]. The proposed degradation mechanism is based on the release behavior observed in the in vitro release study and the mass loss pattern during soil burial. Direct measurements of CEO residues and microbial populations during the degradation process would be needed to further verify this interpretation. Nonetheless, these results suggest that the prepared films exhibit good environmental degradability while balancing short-term functionality with long-term biodegradability.

### 3.7. Antimicrobial Activity

**Figure 6:**
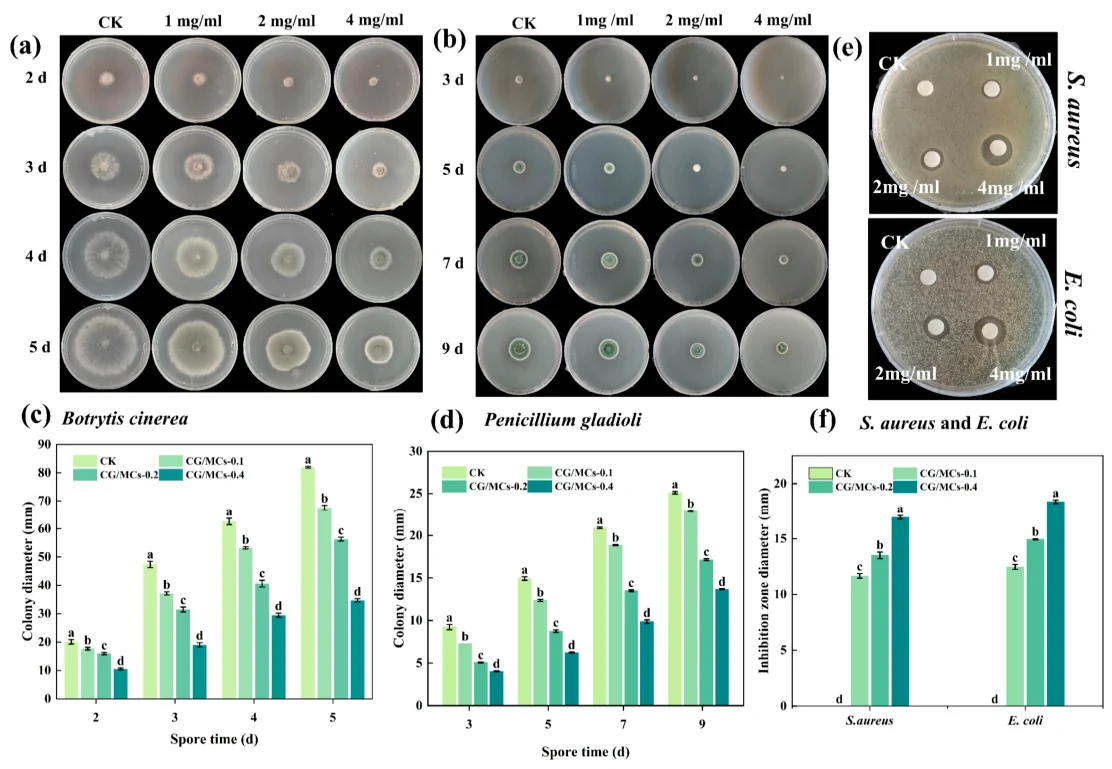
Inhibitory effects of β-CD@CEO MC composite films at different concentrations on *Botrytis cinerea* (**a**,**c**) and *Penicillium gladioli* (**b**,**d**); photos of inhibition zones against *S. aureus* and *E. coli* (**e**); inhibition zone diameters of *S. aureus* and *E. coli* (**f**). Different letters indicate significant differences among treatment groups (*p* < 0.05).

As shown in Figure 6a–d, composite films containing different concentrations of β-CD@CEO MCs exhibited concentration-dependent antifungal activity against both fungi. On day 5, the film with 4 mg/mL MCs reduced the colony diameter against *B. cinerea* from 81.9 mm (CK) to 34.7 mm (57.63% reduction). On day 9, for *P. gladioli*, the diameter decreased from 25.1 mm (CK) to 13.6 mm (47.80% reduction). This effect is consistent with the inclusion and sustained release of active CEO components (e.g., eugenol) by β-CD, which maintains effective concentrations that exert multi-target antifungal actions. Mechanistically, eugenol has been reported to insert its phenolic hydroxyl group into the fungal phospholipid bilayer, causing membrane depolarization and increased permeability [59]. It has also been suggested to target ergosterol through a dual mechanism. It inhibits ergosterol biosynthesis while simultaneously binding directly to membrane ergosterol, thereby disrupting membrane integrity and barrier function [60]. Eugenol has also been reported to induce ROS generation and mitochondrial dysfunction [61]. The differential efficacy against *B. cinerea* versus *P. gladioli* may be related to differences in cell wall architecture. *B. cinerea* possesses a thinner and more permeable cell wall, whereas *Penicillium* species have denser walls with higher chitin content and more extensive β-glucan cross-linking, which provide stronger physical barriers [62,63].

The antibacterial activity of the composite films against *S. aureus* and *E. coli* is shown in Figure 6e,f. They exhibited obvious inhibition zones against both tested bacteria, and the diameters of the inhibition zones increased progressively with increasing film concentration, indicating a concentration-dependent antibacterial activity. At a concentration of 4 mg/mL, the inhibition zone diameters against *S. aureus* and *E. coli* reached 16.93 ± 0.15 mm and 18.37 ± 0.15 mm, respectively, with the former being significantly smaller than the latter. Similar observations have been reported in previous studies, in which Nogueira et al. [64] demonstrated that the antibacterial mechanism of many terpenes and phenylpropanoids is achieved through altering membrane permeability rather than via cell lysis. The phenolic hydroxyl group of eugenol has been shown to disrupt the outer membrane of Gram-negative bacteria by increasing the permeability of the lipopolysaccharide layer, thereby allowing the compound to reach the inner membrane [58]. In contrast, the peptidoglycan layer of *S. aureus* is much thicker than that of *E. coli*, and this thick layer may impede the diffusion of hydrophobic antimicrobial compounds such as eugenol [64,65]. In addition to cell-wall structure, the diffusion behavior of eugenol through the agar matrix could also influence the observed inhibition zone diameters. The larger inhibition zones against *E. coli* may therefore reflect a combination of eugenol’s ability to disrupt the outer membrane, a thinner peptidoglycan layer that allows faster access to the inner membrane, and potentially faster diffusion through the agar matrix, whereas the thick peptidoglycan layer of *S. aureus* may stunt eugenol diffusion despite the absence of an outer membrane. The individual contributions of CEO, β-CD, and the inclusion system to the observed antimicrobial activity were not evaluated separately in the present study. Nevertheless, these results indicate that β-CD@CEO MC composite films show promise as antimicrobial packaging materials for fresh fruit and vegetable preservation.

### 3.8. In Vitro Release Study of Composite Films

**Figure 7:**
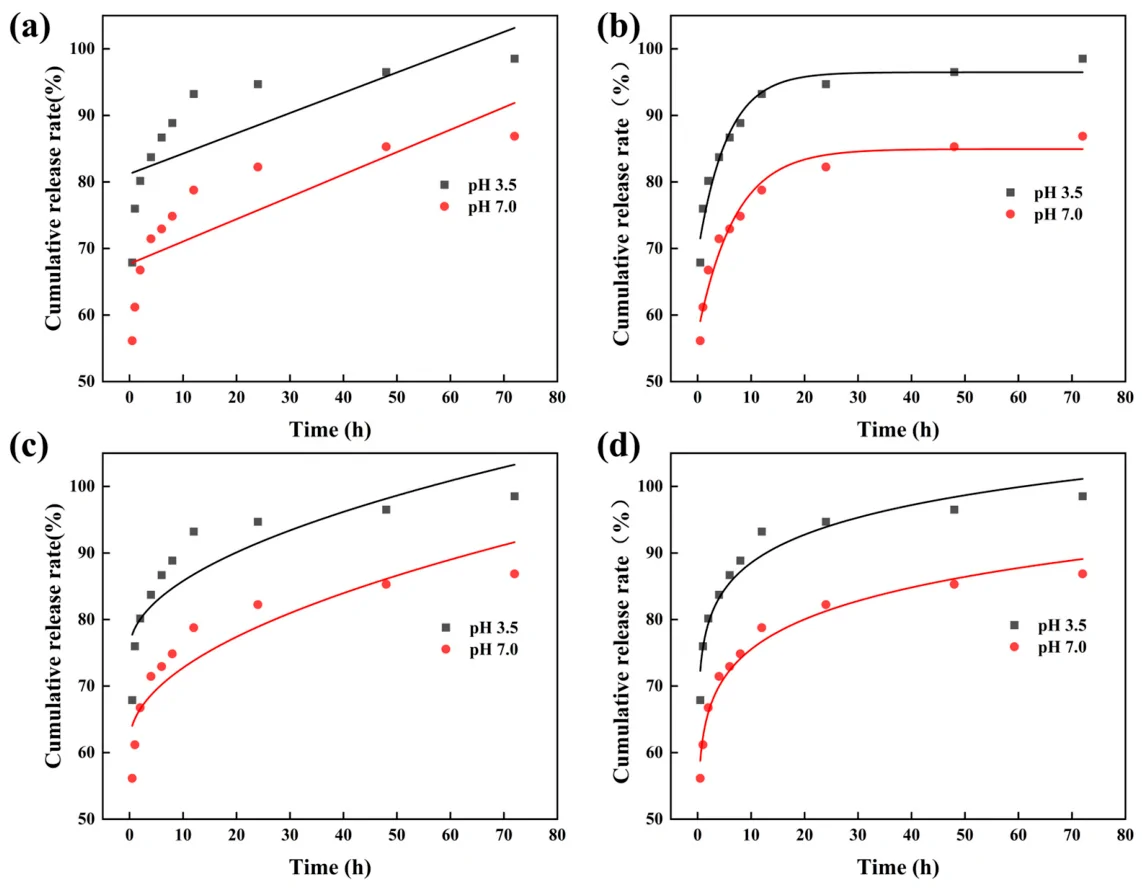
Zero-order (**a**), first-order (**b**), Higuchi (**c**), and Ritger–Peppas (**d**) kinetic models of eugenol release from CG/MCs-0.4 composite films at pH 3.5 and pH 7.0 at 25 °C. Data are shown as mean ± SD (*n* = 3). The corresponding fitting parameters are listed in Table 3.

The cumulative release profiles of eugenol from CG/MCs-0.4 composite films in pH 3.5 acetate buffer and pH 7.0 deionized water at 25 °C are shown in Figure 7. The release behavior exhibited significant pH-dependent characteristics. In the acidic medium at pH 3.5, the release rate of eugenol was remarkably faster than that in the neutral medium at pH 7.0. At 0.5 h, the cumulative release percentages were 67.9% and 56.1% at pH 3.5 and pH 7.0, respectively, indicating an initial burst release in both media. After 72 h, the cumulative release reached 98.5% at pH 3.5, whereas only 87.2% was released at pH 7.0. This initial burst release is functionally beneficial for table grape preservation, as it provides an immediate supply of antimicrobial compounds on the grape surface during the early storage period when fruits are most susceptible to infection, while the subsequent slower phase maintains prolonged protective effects.

To elucidate the release mechanism, the release data were fitted to zero-order, first-order, Higuchi, and Ritger–Peppas kinetic models using the complete 72 h release profiles (Table 3). The results showed that the first-order model exhibited the highest correlation coefficient at pH 3.5 (R^2^ = 0.9496), suggesting that the release of eugenol was primarily driven by the concentration gradient. At pH 7.0, the Ritger–Peppas model provided the best fit (R^2^ = 0.9723), with a release exponent n < 0.5, suggesting a Fickian diffusion-controlled mechanism [66]. The Ritger–Peppas model provides the release exponent (n) to distinguish between Fickian diffusion and anomalous transport, making it suitable for describing the release behavior of the polymer film matrix in this study. This pH-responsive behavior is attributed to the protonation-induced swelling of chitosan chains under acidic conditions, which results in a more porous film structure that facilitates eugenol diffusion from the β-CD cavities [67]. In contrast, under neutral conditions, chitosan maintains a compact conformation, and the release is restricted by molecular diffusion. This biphasic release pattern, consisting of a rapid initial burst followed by a slower sustained phase, enables immediate antimicrobial action on the weakly acidic grape surface while providing prolonged protection, making it a promising strategy for berry fruit preservation. It should be emphasized that the in vitro release test was conducted over a 72 h period, and the 15-day grape preservation outcomes represent the combined effects of the coating’s barrier properties and its antimicrobial and antioxidant activities.

### 3.9. Applications for Table Grape Preservation

**Figure 8:**
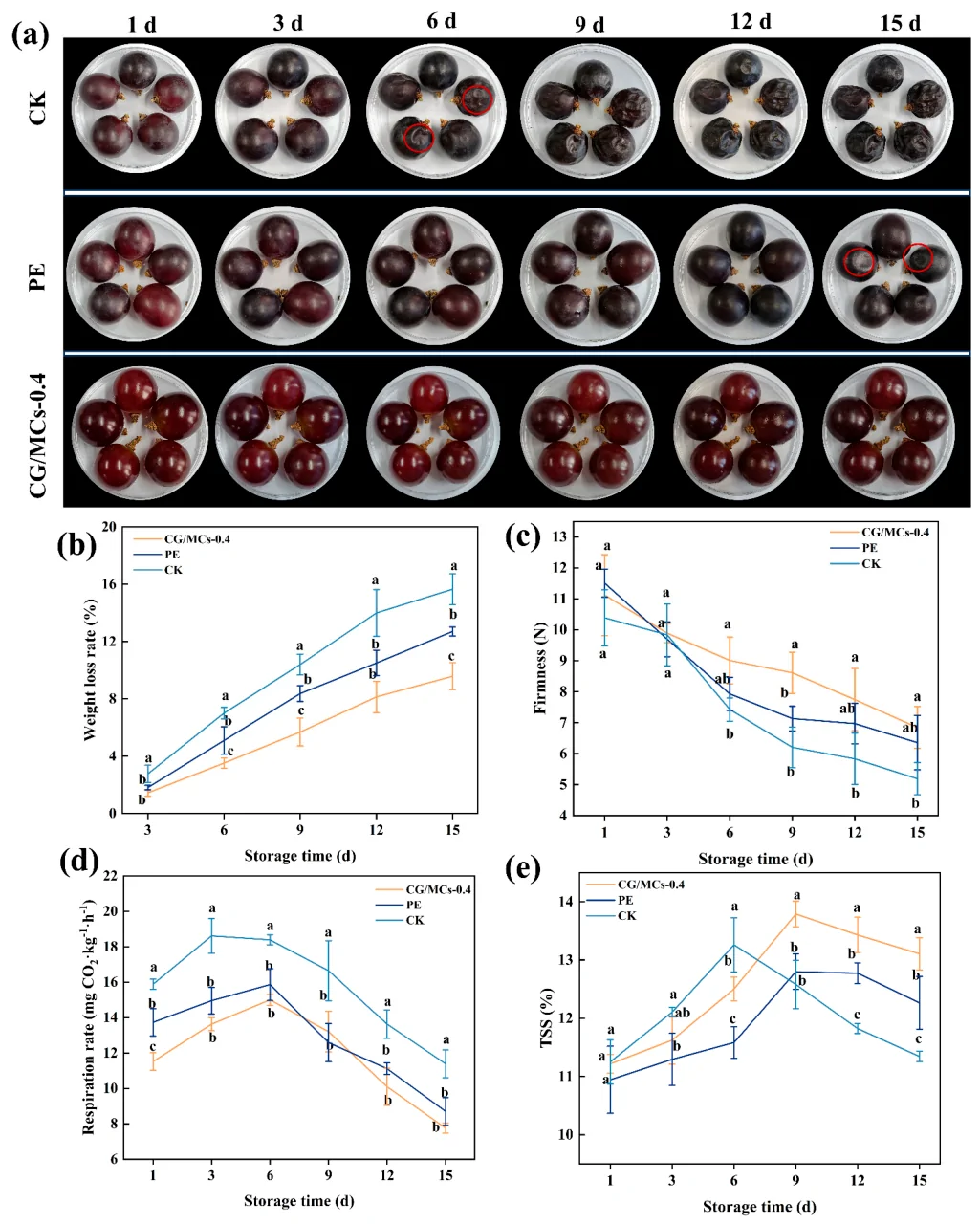
Effects of different treatments on appearance (**a**), weight loss (**b**), firmness (**c**), respiration rate (**d**), and total soluble solids (**e**) of table grapes stored at 4 °C for 15 days. The red circle in (**a**) highlights the visual changes on the grape surface. Different letters indicate significant differences among treatments (*p* < 0.05).

Figure 8a illustrates the visual changes in table grapes during storage. The CK group showed the fastest deterioration, with obvious wrinkling and indentations by day 6, and severe water loss, wilting, and loss of gloss by day 9. The PE group delayed deterioration to some extent, but still showed localized wrinkling and reduced gloss in later stages. In contrast, the CG/MCs-0.4 group maintained plumper berries and bright color throughout storage, with no wrinkling, indentations, or browning, effectively halting postharvest appearance decline and retaining commercial value.

The weight loss rate (Figure 8b) increased gradually with storage time. Throughout storage, the CG/MCs-0.4 group maintained a significantly lower weight loss rate than the PE and CK groups. By day 15, its weight loss was only 9.58%, which was 24.63% and 38.79% lower than those of the PE group (12.71%) and CK group (15.65%), respectively, indicating that the CG/MCs-0.4 composite film effectively reduced fruit water loss. Although the PE film was thicker (42.0 ± 0.7 μm) than the CG/MCs-0.4 coating (27.9 ± 0.4 μm, measured on glass slides) (Supplementary Materials Table S2), the coated grapes exhibited lower weight loss than the PE-packaged ones. This difference likely reflects a combination of barrier and active functional contributions; however, the two packaging configurations differed in geometry and mass-transfer conditions, and the actual coating thickness, coverage, and uniformity on the curved grape surface were not directly quantified, making it difficult to deconvolute the relative contributions of barrier properties versus antimicrobial/antioxidant activities to the observed weight loss reduction.

Fruit firmness is a key indicator of textural quality. As shown in Figure 8c, the firmness of all three groups decreased continuously during storage, with the CK group softening the fastest. By day 15, firmness had decreased to 5.19 N (CK), 6.36 N (PE), and 6.84 N (CG/MCs-0.4). The postharvest decline in fruit firmness is attributed to cell wall pectin degradation and water loss. The CG/MCs-0.4 coating delayed firmness decline by creating a micro-controlled atmosphere and reducing water transpiration.

Respiration rate is a key physiological indicator of fruit metabolic activity. As shown in Figure 8d, the respiration rates of all three groups exhibited a typical trend of first increasing and then decreasing. The CK group peaked on day 3, whereas the PE and CG/MCs-0.4 groups did not reach their peaks until day 6. After 15 days of storage, the respiration rate of the CK group was 11.39 mg CO_2_·kg^−1^·h^−1^, while the rates of the PE and CG/MCs-0.4 groups decreased to 8.70 and 7.75 mg CO_2_·kg^−1^·h^−1^, respectively, representing reductions of 23.62% and 31.96% compared with the CK group. Both treated groups maintained lower respiration rates during the middle and late storage stages. The delayed respiration peak in the CG/MCs-0.4 group may be related to gas barrier properties and antimicrobial activity of the coating. The coating is expected to form a semi-permeable barrier on the grape surface that reduces O_2_ permeability and slows oxidative metabolic processes, as reported for chitosan-based coatings in fruit preservation [1,68]. Chitosan itself has been reported to reduce respiration rates by decreasing O_2_ uptake and suppressing key respiratory enzymes such as succinate dehydrogenase. It is also possible that CEO released from the β-CD microcapsules contributes to inhibiting surface microbial growth [10,52], which may prevent microbial-induced respiratory stimulation. Overall, these multiple pathways are likely to operate jointly, and the observed delay in the respiration peak is consistent with the combined effects of the coating’s barrier and antimicrobial properties.

During storage, TSS content first increased and then decreased (Figure 8e). The CK group peaked at 13.26% on day 6 and then declined rapidly. The PE group reached 12.80% on day 9 and then decreased slowly. The CG/MCs-0.4 group maintained significantly higher TSS levels than the CK group throughout storage, peaking at 13.79% on day 9 with the smallest decline in later stages, indicating that the CG/MCs-0.4 coating effectively maintained fruit nutritional and flavor quality by suppressing respiration and reducing soluble sugar loss.

**Figure 9:**
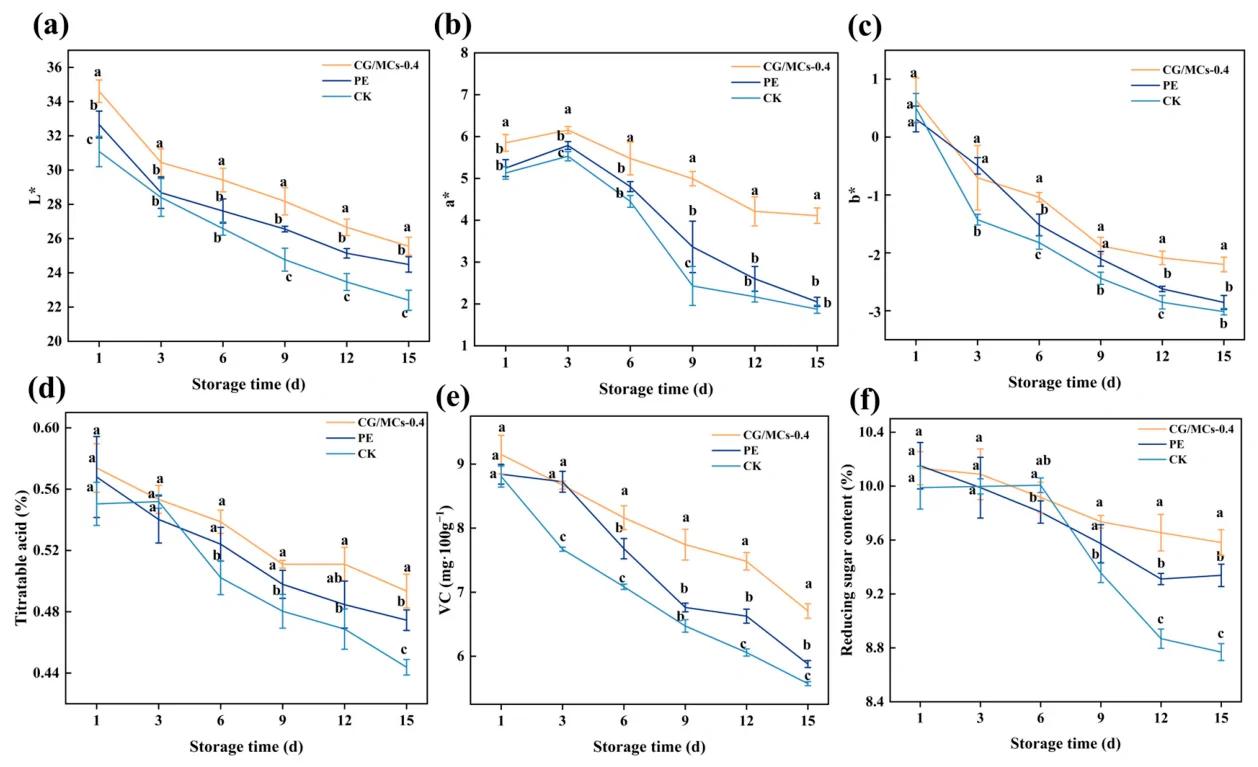
Effects of different treatments on L* (**a**), a* (**b**), b* (**c**), titratable acidity (**d**), vitamin C (**e**), and reducing sugar (**f**) contents of table grapes stored at 4 °C for 15 days. Different letters indicate significant differences among groups (*p* < 0.05).

The chromaticity parameters (L*, a*, b*) were used to monitor changes in grape skin color (Figure 9a–c). During storage, L* values of all groups decreased continuously, with the CG/MCs-0.4 group remaining consistently higher than the PE and CK groups. After 15 days, the L* values were 25.55 (CG/MCs-0.4), 24.49 (PE), and 22.40 (CK). The a* values firstly increased and then decreased, reaching 4.11, 2.05, and 1.87, respectively, after 15 days. The b* values shifted negatively overall, with the CG/MCs-0.4 group remaining higher than the other two groups throughout storage. Collectively, the CG/MCs-0.4 coating maintained the postharvest skin color quality of table grapes by regulating pigment metabolism.

Titratable acidity is an important indicator of total organic acid content in fruit, reflecting flavor quality and ripeness. During storage, titratable acidity decreased continuously in all groups (Figure 9d). By day 15, the titratable acidity in the CG/MCs-0.4, PE, and CK groups had decreased to 0.49%, 0.47%, and 0.44%, respectively, with the CG/MCs-0.4 group remaining significantly higher than the PE and CK groups throughout storage.

Vitamin C (VC) content decreased continuously during storage (Figure 9e), primarily due to auto-oxidation, respiratory metabolism, and reactive oxygen species accumulation. From day 3 onward, the CG/MCs-0.4 group maintained significantly higher VC levels than the CK group. By day 15, VC content had declined to 6.71, 5.88, and 5.57 mg/100 g in the CG/MCs-0.4, PE, and CK groups, respectively. Notably, the CG/MCs-0.4 group showed VC levels 12.37% and 16.99% higher than those in the PE and CK groups. The enhanced VC retention in the CG/MCs-0.4 group may be related to the strong radical scavenging activity of the film, which has been reported to neutralize ROS and reduce oxidative VC degradation [69], as well as its gas barrier properties that limit O_2_-dependent oxidation, and the antimicrobial activity of CEO that has been shown to prevent microbial consumption of VC [10,52]. These three functions together provide a plausible explanation for the improved VC retention.

Changes in reducing sugar content are shown in Figure 9f. During storage, reducing sugar content in the CK group briefly increased in the early stage (days 1–6) and then declined, whereas the CG/MCs-0.4 and PE groups showed a gradual decrease. On day 15, reducing sugar contents were 8.77% (CK), 9.34% (PE), and 9.58% (CG/MCs-0.4). The initial increase in the CK group was attributed to higher hydrolase activity and a concentration effect due to water loss. These results indicate that the CG/MCs-0.4 coating effectively maintains higher reducing sugar levels.

## 4. Conclusions

In this study, a biodegradable composite active packaging film (CG/β-CD@CEO MCs) with pH-responsive sustained release functionality was developed. The incorporation of β-CD@CEO microcapsules significantly enhanced the mechanical strength, UV shielding, and water vapor barrier properties of the chitosan/gelatin film matrix. The film exhibited strong antioxidant and broad-spectrum antimicrobial activities, effectively inhibiting the growth of both fungi (*B. cinerea* and *P. gladioli*) and bacteria (*S. aureus* and *E. coli*). Notably, the pH-responsive release behavior of CEO enabled faster release under acidic conditions, which is particularly advantageous for application on the weakly acidic surface of table grapes. In grape preservation trials, the CG/MCs-0.4 film significantly reduced weight loss, maintained firmness and color, and preserved key nutritional components compared to commercial PE packaging and untreated controls. These findings suggest that the integration of β-CD encapsulation with a chitosan/gelatin film matrix represents a promising strategy for designing active packaging materials with controlled release functionality. Future work should focus on optimizing the balance between water resistance and biodegradability to further enhance the practical applicability of the films. Additionally, the preservation performance of this system on other perishable fruits and vegetables warrants investigation.

## Figures and Tables

**Table 1:** Drug loading and encapsulation efficiency of microcapsules (*n* = 3).

Sample	Drug Loading DL (%)	Encapsulation Efficiency EE (%)
1	5.68	36.90
2	6.20	40.25
3	5.98	38.85
Mean ± SD	5.96 ± 0.26	38.67 ± 1.68

**Table 2:** Color, Thickness, and Opacity of CG/β-CD@CEO Composite Films.

Category	L*	a*	b*	ΔE	Thickness (mm)	Opacity
CK	93.1 ± 0.34 ^a^	0.06 ± 0.03 ^b^	4.54 ± 0.08 ^c^	6.15 ± 0.25 ^d^	0.022 ± 0.001 ^c^	1.94 ± 0.05 ^a^
CG/MCs-0.1	91.39 ± 0.78 ^b^	0.07 ± 0.03 ^b^	5.65 ± 0.04 ^a^	8.12 ± 0.54 ^c^	0.022 ± 0.001 ^c^	1.97 ± 0.07 ^a^
CG/MCs-0.2	87.48 ± 0.38 ^c^	0.18 ± 0.04 ^a^	5.26 ± 0.08 ^b^	10.99 ± 0.35 ^b^	0.025 ± 0.001 ^b^	2.03 ± 0.09 ^a^
CG/MCs-0.4	82.05 ± 0.77 ^d^	0.24 ± 0.09 ^a^	5.72 ± 0.17 ^a^	16.09 ± 0.66 ^a^	0.027 ± 0.001 ^a^	3.59 ± 0.08 ^b^

Different letters indicate significant differences among groups (*p* < 0.05).

**Table 3:** Kinetic fitting parameters of eugenol release from CG/MCs-0.4 composite films at pH 3.5 and pH 7.0.

Kinetic Model	pH	Fitting Formula	Fitted Equation	*R* ^2^
Zero-order	3.3 7.0	*y* = *ax* + *b*	*y* = 0.304*x* + 81.214 *y* = 0.336*x* + 67.684	0.4985 0.5845
First-order	3.5 7.0	*y* = *a* × (1 − *e*^(−*b* ×^ *^x^*^)^)	*y* = 96.480 × (1 − e^−0.18*x*^) *y* = 84.940 × (1 − e^−0.14*x*^)	0.9496 0.9573
Higuchi	3.5 7.0	*y* = *a* × *x*^1/2^ + *b*	*y* = 3.287 × *x*^1/2^ + 75.391 *y* = 3.547 × *x*^1/2^ + 61.525	0.7252 0.8023
Ritger–Peppas	3.5 7.0	*y* = *a* × *x^b^*	*y* = 75.751 × *x*^0.068^ *y* = 62.270 × *x*^0.084^	0.9371 0.9723

## Data Availability

The original contributions presented in this study are included in the article/Supplementary Materials. Further inquiries can be directed to the corresponding author.

## Supplementary Materials

The following supporting information can be downloaded at https://www.mdpi.com/article/10.3390/foods15172957/s1. Text S1: Characterization of β-CD@CEO MCs and composite films; Text S2: Antimicrobial activity assays (antifungal and antibacterial); Text S3: Determination of drug loading in composite films (including Table S1); Text S4: Grape treatment and storage conditions.
