# Supplementary material for: Biodegradable Chitosan Films Incorporated with β-Cyclodextrin Microcapsules Loaded Clove with Essential Oil for Table Grape Preservation
Source: Foods. 2026 Aug 22;15(17):2957. doi: 10.3390/foods15172957 (PMC13564530; doi:10.3390/foods15172957)
Supplement: Supplementary file 1 [file foods-15-02957-s001.zip › foods-4481997-supplementary.pdf]

Supplementary Material

S1.Characterization of β-CD@CEO MCs and Composite Films

1.1 Characterization of β-CD@CEO MCs

The chemical structures of β-CD, CEO, and β-CD@CEO MCs were characterized by Fourier transform infrared (FTIR) spectroscopy (Nicolet iS50, Thermo Fisher Scientific, USA) using the KBr pellet method over the range of 4000–400 cm^{-1} at a resolution of 4 cm^{-1}. Thermal decomposition properties of the films were evaluated using a TG-DSC thermal analyzer (STA 449 F5, NETZSCH, Germany). Thermal stability of β-CD and β-CD@CEO MCs was analyzed under a N_2 atmosphere from 30 °C to 800 °C at a heating rate of 10 °C/min. Surface morphology was observed by scanning electron microscopy (SEM) (S-3400N, Hitachi, Japan). Samples were mounted on aluminum stubs with conductive adhesive, sputter-coated with gold, and observed at an accelerating voltage of 5.0 kV. Crystal structures of β-CD and β-CD@CEO MCs were determined by X-ray diffraction (XRD) (D8 Advance, Bruker AXS, Germany) over a 2θ range of 10°–80°.

1.2. Characterization of CG/β-CD@CEO Composite Films

The same methods described in S1.1 were applied to characterize the CG/β-CD@CEO composite films.

S2. Antimicrobial Activity

2.1 Antifungal activity

The antifungal activity of the composite films was evaluated using the poisoned agar method Chen et al [22]. First, PDA media containing the film-forming solution (with β-CD@CEO MCs) at different concentrations were prepared. The plates were then inoculated with *Botrytis cinerea* and *Penicillium gladioli*, respectively. A 6-mm diameter *B. cinerea* disc (cut from the colony edge using a sterile punch) was placed in the center of the PDA plate; Then, 10 μL of *P. gladioli* spore suspension (1×10^6 CFU/mL) was pipetted onto the PDA plate. The plates were incubated in the dark at 26 °C, and colony diameters were measured daily using the cross-counting method. Three plates were used per concentration group, and the experiment was performed in

triplicate.

2.2 Antibacterial activity

Antibacterial activity was evaluated using the disk diffusion method (Kirby–Bauer method), following the procedure described by Wang et al [23]. The test organisms were *Staphylococcus aureus* and *Escherichia coli*. Overnight bacterial cultures were diluted to 1.0×10^6 CFU/mL, and 100 μL was evenly spread onto the surface of LB agar plates. Sterile filter paper discs (8 mm in diameter) were immersed in a composite membrane solution containing different concentrations of microcapsules for 30 min, then removed with sterile forceps and placed on the inoculated plates. Filter paper discs moistened with sterile water served as the control group. The plates were inverted and incubated at 37 °C for 24 h. After incubation, the diameter of the inhibition zone (mm) was measured using a Vernier caliper. Each sample was tested in triplicate, and results are expressed as the mean ± standard deviation.

S3 Determination of Drug Loading in Composite Films

The eugenol loading of CG/MCs-0.4 composite films was determined by UV-visible spectrophotometry, according to the method described by Huang et al [18]. with slight modifications. The dried CG/MCs-0.4 composite films were cut into small pieces, and 100 mg of the film fragments were accurately weighed and placed into a conical flask. Then, 10 mL of anhydrous ethanol was added, and the mixture was ultrasonicated for 45 min to disrupt the β-CD@CEO microcapsules within the film matrix and completely release the encapsulated eugenol into the solvent. During the extraction process, anhydrous ethanol was supplemented to a final volume of 35 mL due to the swelling of the film fragments. The extract was transferred to a centrifuge tube and centrifuged at 4,000 r/min for 10 min. The supernatant was collected, and its absorbance was measured at 280 nm. Blank CG films (without microcapsules) were processed in the same manner as controls to subtract the background absorbance of the film matrix. The concentration of eugenol in the extract was calculated using a eugenol standard curve ($y = 0.0273x - 0.0069$, $R^2 = 0.9998$). All samples were measured without dilution. The drug loading of the composite films was calculated using the following formula:

$$DL(\%) = \frac{C \times V}{M_{film}} \times 100\%$$

Where C is the mass concentration of eugenol in the extract (μg/mL), V is the total volume of the extract (35 mL), and M film is the mass of the composite film sample (100 mg). All results are expressed as eugenol equivalents.

Table S1. Drug loading of CG/MCs-0.4 composite films (n = 3)

Sample	Drug loading DL (%)
1	0.386
2	0.385
3	0.379
Mean±SD	0.383±0.004

S4 Grape Treatment and Storage Conditions

Kyoho grapes of uniform size and free from mechanical damage were selected, with stems left intact. The grapes were sterilized in 0.2% (v/v) sodium hypochlorite solution for 5 min, rinsed with sterile water, and air-dried. The table grapes were then randomly divided into three groups: control (CK), polyethylene (PE) packaging, and CG/MCs-0.4 coating. For the CG/MCs-0.4 group, the grapes were immersed in the film-forming solution for 1 min, then removed, hung, and air-dried at room temperature (25 °C) for 30 min to form a uniform film. This immersion-drying process was repeated once (two immersions in total) to achieve an uniform coating thickness. Grapes in the PE group were placed in polyethylene bags and sealed, while the CK group was no treatment. All fruits were placed in plastic containers (5 per container) and stored at 4 °C and 90% relative humidity. Observations were performed and quality indicators were measured every 3 days.

Table S2. Thickness of CG/MCs-0.4 coating and PE film

Sample	Thickness (μm)
CG/MCs-0.4 coating	27.9 ± 0.4
PE film	42.0 ± 0.7

18. Huang, J.L.; Pu, J.R.; Yang, Z.B.; Zhang, S.Y.; Zhang, Z.H.; Lu, Q.; Song, D.; Li, X.; Fang, Z.F.; Liu, Y.T.; et al. Development of a Syzygium aromaticum, L. essential oil/ hydroxypropyl-β-cyclodextrin inclusion complex: Preparation, characterization, and evaluation. *Industrial Crops and Products* **2024**, *214*, 118500.

22. Chen, C.; Li, Z.J.; Yu, H.Y.; Su, L.B.; Tian, H.X.; Yuan, H.B. Effects of sustained release of tea polyphenols from a bacterial cellulose-based coating to prolong shelf-life of fresh walnuts. *Food Chemistry* **2026**, *520*, 149795.

23. Wang, F.; Wang, R.H.; Pan, Y.J.; Du, M.; Zhao, Y.; Liu, H.Q. Gelatin/Chitosan Films Incorporated with Curcumin Based on Photodynamic Inactivation Technology for Antibacterial Food Packaging. *Polymers* **2022**, *14*, 1600.
